# iRFP (near-infrared fluorescent protein) imaging of subcutaneous and deep tissue tumours in mice highlights differences between imaging platforms

**DOI:** 10.1186/s12935-021-01918-8

**Published:** 2021-05-03

**Authors:** C. Hall, Y. von Grabowiecki, S. P. Pearce, C. Dive, S. Bagley, P. A. J. Muller

**Affiliations:** 1 Tumour Suppressors Group, CRUK Manchester Institute, University of Manchester, Alderley Park, Manchester, SK10 4TG UK; 2 Cancer Biomarker Centre, CRUK Manchester Institute, University of Manchester, Alderley Park, Manchester, SK10 4TG UK; 3 Visualisation, Irradiation and Analysis, CRUK Manchester Institute, University of Manchester, Alderley Park, Manchester, SK10 4TG UK

**Keywords:** IRFP, Imaging, Mice, Tumours

## Abstract

**Background:**

In vivo imaging using fluorescence is used in cancer biology for the detection, measurement and monitoring of tumours. This can be achieved with the expression of fluorescent proteins such as iRFP, which emits light at a wavelength less attenuated in biological tissues compared to light emitted by other fluorescent proteins such as GFP or RFP. Imaging platforms capable of detecting fluorescent tumours in small animals have been developed but studies comparing the performance of these platforms are scarce.

**Results:**

Through access to three platforms from Xenogen, Bruker and Li-Cor, we compared their ability to detect iRFP-expressing subcutaneous tumours as well as tumours localised deeper within the body of female NSG mice. Each platform was paired with proprietary software for image analyse, but the output depends on subjective decisions from the user. To more objectively compare platforms, we developed an ‘in house’ software-based approach which results in lower measured variability between mice.

**Conclusions:**

Our comparisons showed that all three platforms allowed for reliable detection and monitoring of subcutaneous iRFP tumour growth. The biggest differences between platforms became apparent when imaging deeper tumours with the Li-Cor platform detecting most tumours and showing the highest dynamic range.

**Supplementary Information:**

The online version contains supplementary material available at 10.1186/s12935-021-01918-8.

## Introduction

Precise imaging and serial measurement of mouse tumours is important across a range of cancer research projects. For palpable, subcutaneous tumours, calliper measurements (in which the length and diameter of the tumour gives an estimate of its volume) is one of the most used methods for routine assessment of tumour progression [1, 2]. For deeper tumours that are not palpable, imaging techniques such as MRI, X-ray, fluorescent microscopy or luminometry are used [3]. Of these, the most commonly used technique in preclinical research is luminometry which requires an enzymatic luciferase reaction for visualisation. This technique can be used on injected cells expressing constitutive or inducible luciferase, in genetically engineered mouse models or using injected viruses that carry the luciferase gene. In practice, the d-luciferin substrate is usually injected in the scruff of the mouse and allowed to circulate for 5–10 min while the mouse is active before anaesthesia, after which images can be acquired using a CCD based imaging platform. The drawback of this approach is the requirement for d-luciferin injection, which adds an invasive element to an otherwise non-invasive technique. The researcher then has to allow time for the production of luminescence through the luciferase reaction. The optimal time to measure tumour size and location within the animal is within ~ 10 min of the injection [4, 5], but variations in how much d-luciferin is injected or diffuses into the bloodstream to reach the tumour, and how much time elapses before imaging can lead to variation in measurements. Timing d-luciferin injections so that every animal is measured at the same time after administration, especially if multiple animals are measured at the same time, can be challenging and time consuming. This variability has been shown to have a significant amount of change in luminescence output in the early time period after injection [4, 5]. However, used carefully, luciferase can be an excellent imaging tool with low background and a very high sensitivity in detecting tumours.

Fluorescent proteins, such as GFP (green fluorescent protein, 510 nm emission peak), mCherry (610 nm emission peak [6]) and mStrawberry (596 nm emission peak [6]) are generally stably or inducibly expressed in the cell lines from which a tumour can be established. Similarly to luciferase, they may also be induced using viruses carrying the fluorescent protein’s coding sequence. A setback of using fluorescence is autofluorescence in animals, caused by tissues or the diet [7]. Low-autofluorescence feed is available and has been demonstrated to reduce background to some extent [7]. Luciferase signal is usually stronger than fluorescent signals, due to its typically longer capture period, lack of photobleaching and wider imaging spectrum, allowing detection of weaker or attenuated signals (through hair, pigmentation, or tumour depth). For this reason, D-luciferin is often used in studies where the signal may be weak or located deep within the body. Like other fluorescent proteins, the near-infrared fluorescent protein iRFP avoids the variability due to dose and time factors of d-luciferin injections, as well as welfare concerns related to the additional injection. As its wavelength falls outside the absorption spectrum of haemoglobin and oxy-haemoglobin (found extensively in mammalian tissues), the iRFP signal is less attenuated in tissues and therefore has better signal to background ratios than other fluorescent proteins [8].

There are a number of iRFP proteins, but the first and most widely used is iRFP713, which has an emission peak at 713 nm [9]. Infrared proteins utilise biliverdin as a chromophore and iRFP713 requires endogenous biliverdin to become fluorescent [9]. iRFP713 has been used to image single cells as well as organs and tumours in whole organisms in real time [9–12]. Various research groups have successfully used iRFP to monitor tumour growth or to detect and monitor formation of metastasis [9, 12–20]. Other iRFP variants exist (iRFP670, miRFP680, iRFP682, iRFP702 and iRFP720), which all have slightly differing excitation and emission spectra [21–23].

Here, we directly compare the performance of three in-vivo imaging platforms to track tumours over a two-week time period. We used the Xenogen VivoVision IVIS 200 (XVI), Bruker In-Vivo Xtreme (BIX) and the Li-Cor Pearl Trilogy (LPT) to examine growth of subcutaneous tumours and deep tissue tumours arising from tail vein injections of iRFP-expressing cancer cell lines. All platforms are able to measure subcutaneous iRFP tumours reliably, but deep-tissue tumours were detected to variable degrees by each of the three platforms, with the LPT detecting most tumours.

## Results

### Determining the dynamic range and signal linearity of all platforms

**Fig. 1:**
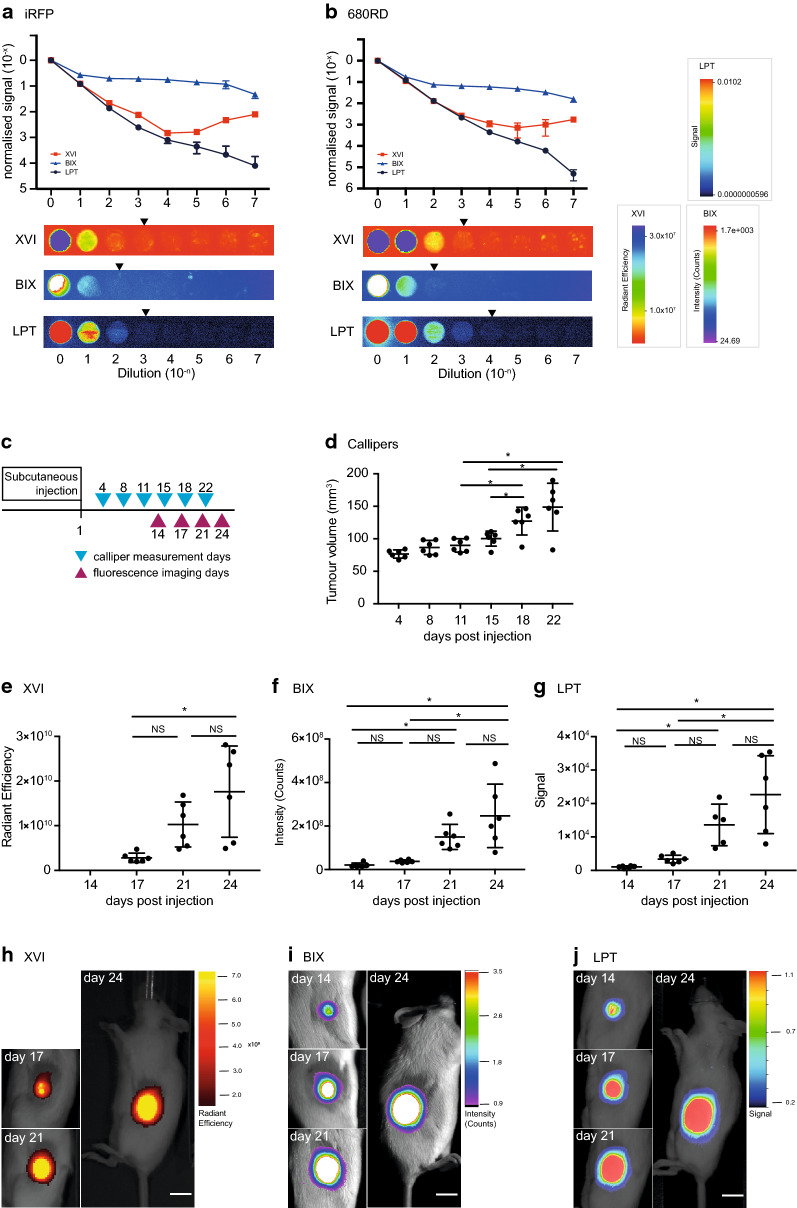
**a**, **b** Normalised signal of a logarithmic dilution of fixed iRFP expressing A431 cells (**a**) or iRFP antibody (680RD) (**b**), suspended in low-melt agarose, and a representative example of each dilution series as seen by each platform. Gating was performed to be able to see the lowest possible dilution above background. A representative example of one of the triplicates imaged in each platform is shown below the graph. Arrows represent the subjective last visible dilution using gating optimised to see the lowest possible signal distinguishable from background. LPT = Li-Cor Pearl Trilogy, BIX = Bruker In-Vivo Xtreme, XVI = Xenogen VivoVision IVIS 200. Quantification was performed in each platform’s own software and results of technical triplicates with standard deviation error bars are shown. Points without error bars have an error too low to be rendered in Prism. **c** Schematic representation of calliper measurements (blue triangles) and imaging measurements (green triangles) in days after subcutaneous injection of iRFP expressing H1299 cells. **d** Tumour volume over time as measured by callipers. Error bars represent ± SD, (asterisk indicates Tukey’s multiple comparisons test p < 0.05, NS p > 0.05). **e**–**g** Measurements of fluorescent intensity of tumours over time as measured by the XVI (fluorescent intensity: radiant efficiency) (**e**) BIX (fluorescent intensity: counts) (**f**) or the LPT (fluorescent intensity: signal) (**g**). Error bars represent ± SD, (asterisk indicate statistically significant differences, ANOVA, Tukey’s multiple comparisons test p < 0.05, NS indicates no significant difference). **h**–**j** Images of the tumour of mouse 2 on each measurement day for the XVI (**h**), BIX (**i**) and LPT (**j**). Images show fluorescent intensity as shown by each platform’s software (false colours mode) overlaid on brightfield images, to show example of tumour growth. Scale bars for each software are presented. XVI was gated to day 17, BIX and LPT were gated to day 14

In order to test the dynamic range of the three different imaging systems: Xenogen VivoVision IVIS 200 (XVI), Bruker In-Vivo Xtreme (BIX) and Li-Cor Pearl Trilogy (LPT), we created a point source test, mimicking a subcutaneous tumour-like situation in which we could control the amount of fluorescence in a linear manner. We generated a 10 × dilution series of iRFP-expressing A431 cells as a representative for iRFP-expression (these cells express high levels of iRFP, Fig. 1a) or a near-infrared secondary antibody (680RD) with similar excitation and emission spectra to iRFP (Fig. 1b) by dilution in agarose in a 96-well plate. The dilutions aimed to be in the low-end of detection range, so as to best find a limit of detection for small tumours. Of all machines, the LPT detects the most linear range with the iRFP-expressing cells or the 680RD-coupled antibody. Of note, the LPT saves images at 22-bit while the other platforms use 16-bit (Fig. 1a, b). However, it should be noted that the lowest visible dilution against background when using false colours is the same for both the LPT and the XVI (Fig. 1a) and only marginally better in the LPT for the antibody dilution (Fig. 1b). This is important as decisions (gating and tumour delineation) are usually taken using subjective visual clues when analysing tumours in mice. Before testing the performance of the platforms with mice, we next wanted to determine if the signal was stable or subject to photobleaching by repeated measurements. We therefore measured the test plate again in the LPT after all tests and acquisitions were completed on the other platforms. Even after multiple test exposures between 5 and 60 s were performed on all platforms, the results indicate that there is no qualitative difference between the first or final readings, and that photobleaching of iRFP caused by repeated measures would not cause differences between platform measurements (Additional file 1: Fig. S1A).

### iRFP signals in subcutaneous tumours

H1299 cells stably expressing iRFP were injected subcutaneously into mice. The volume of resulting tumours was estimated using calliper measurements, and imaged for infrared fluorescence over time as indicated in Fig. 1c. Subcutaneous tumour growth was followed using calliper measurements from the first day that tumours were palpable (day 4 after injection) and then biweekly. These measurements showed statistically significant increases in growth from day 15 to day 18, but not from day 18 to day 22 (Fig. 1d).

**Fig. 2:**
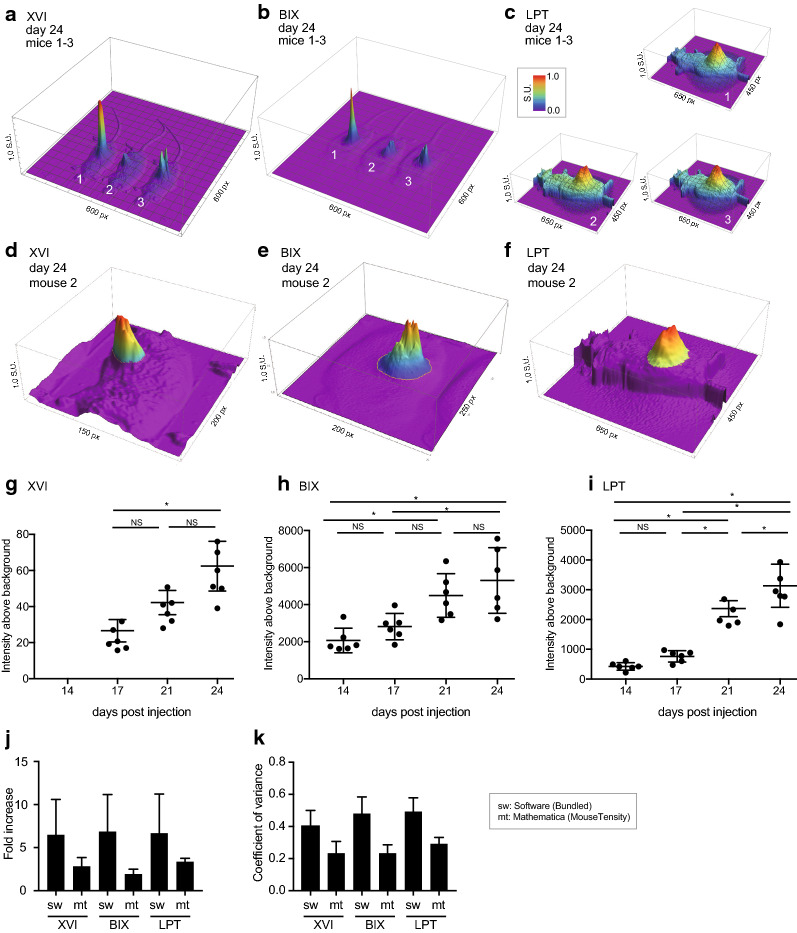
**a**–**c** Mouse tumour intensity outputs from MouseTensity of data captured on the XVI (**a**), the BIX (**b**) or the LPT (**c**) on day 24. Mouse 1, 2 and 3 are shown, grouped by cage in the XVI and the BIX. Mouse 1, 2 and 3 are shown separately as the images are acquired per mouse in the LPT. The highest intensity in each image is normalised to 1. (S.U.: Standardised Unit) A colour scale indicating highest and lowest values in indicated on the top right of A. **d**–**f** Representative example of mouse 2 on day 24. Data from XVI (**d**), BIX (**e**) or LPT (**f**) are visualised (S.U.: Standardised Unit). The section coloured rainbow is used as the measurement signal, while the monochrome purple is considered noise. The difference is delineated by a yellow line. **g**–**i** Measurements of fluorescent intensity of tumours over time as measured by the XVI (**g**) the BIX (**h**) or the LPT (**i**). Error bars represent ± SD, (ANOVA: asterisk indicates Tukey’s multiple comparisons test p < 0.05, NS p > 0.05). **j** Fold increase from average tumour size on day 17 to average tumour size on day 24. Measurements were calculated with each platform’s software. Error bars represent + SD. **k** Coefficients of variance for the software provided with each platform. Coefficient of variance was measured as a function of the final day. Error bars represent + SD

iRFP was imaged on the days preceding the calliper measurements. On all three different imaging platforms, mice were positioned laterally so that the tumour faced the camera. Specifications and differences between machines are listed in Additional file 2: Tables S1 and S2. To determine background, we quantified the fluorescence of an area of the mouse without tumour between the neck and upper torso, which was highly similar in all mice and all time points. Since the LPT displayed the highest dynamic range (Fig. 1a, b), we examined how background changed across a mouse and over time on this platform. Contrary to the XVI and BIX, the LPT was able to clearly separate the mouse background from the basal background of the imaging bed (visible in Fig. 2c, f). Of note, this results in a lower apparent tumour signal in the 3D representations, because the mouse background is clearly above basal background. The 3D representations per mouse highlight that the mouse background signal is uniform in all platforms. In addition, the background does not change over the four measuring days (Additional file 1: Fig. S1B).

A significant increase in tumour growth from day 17 to day 24 was seen and measured on all platforms (Fig. 1e–j) (due to incorrect set up of filters on the XVI, measurements of day 14 could not be used). Absolute fluorescence intensity was highly variable between platforms since each platform’s analysis software has a different method for calculating and displaying fluorescence intensity. To illustrate objective differences between platforms, we exported the raw TIFF files from each platform and plotted the data into a 3D model in Mathematica (MouseTensity) where Z-height is based on fluorescent intensity. The resulting images from three mice imaged on day 24 with each platform are shown in Fig. 2a–c. The 3D nature of the projection shows background and signal at the same time, and this allows users to more effectively and more intuitively select a region of interest as shown for mouse 2 at day 24 (Fig. 2d–f). 3D projections obtained from all mice on day 17 and day 24 from the raw signal are shown in Additional file 1: Figs. S2 and S3. After selecting tumour boundaries, guided by the 3D representation, we then calculated the intensity in each tumour and plotted these in a similar manner to Fig. 1e–g (Fig. 2g–i).

Despite the varying absolute fluorescent intensities between different platforms, a similar fold increase of about 6-fold in tumour growth over time (day 17–24) was measured using all platforms, regardless of the analysis used (Fig. 2j). This fold increase was generally lower, 2.5-fold, using Mathematica than the proprietary software. Furthermore, signal intensity measurements using Mathematica have a lower variation (Fig. 2k) than observed using propriety software programs (Fig. 2k) as measured by the coefficient of variance (Fig. 2k). The consequence of this finding was that the increased tumour growth between days 17–21, previously non-significant (at a 95% confidence interval) in the LPT (Fig. 1g), was significant at a 95% confidence interval when the images were reanalysed with Mathematica and p-values were generally smaller (Fig. 2i; Additional file 2: Table S3. The lower fold-changes using Mathematica for days 17–21 (XVI: 1.8x, BIX: 1.7x, LPT: 2.4x) compared to proprietary software (XVI: 3.7x, BIX: 4.1x, LPT: 3.6x) were more consistent with the calliper volume estimations for days 18–22 (1.5 ×) and may provide a more linear comparative tumour size estimate. These analyses also suggest that images taken using each platform hold similar information and are more comparable across platforms in terms of variation or signal to noise ratios than was expected. Overall, these data infer that for superficial tumours, any of these machines is well suited to study growth rate in time.

Notably, the brightest portion of the tumour images in the XVI sometimes clipped the upper limits of detection (Additional file 1: Fig. S4A). In retrospect, the default “medium” binning setting on the XVI was misleading to the user as in reality this can be considered high (8 × 8 binning). This binning results in a multiplication of the signal intensity by 64 compared to no binning, enhancing the potential of signal clipping. Of note, the lowest possible binning setting for the XVI is 4 × 4. Each platform was set up on the first day to measure tumours with identical settings used on each following day, in order to enable comparison of all intensities over that time period. The dynamic range remained unsaturated for both the LPT and the BIX platforms, possibly accounting for the highest fold increases observed.

When comparing tumour growth in individual mice, the LPT was more similar to the XVI (Additional file 1: Fig. S4B–D), consistently showing the lowest growth in mice 2 and 5, and fastest tumour growth in mice 1, 4 and 6. Although the BIX measurements showed the same trend, mouse 6 measurements were substantially higher throughout the experiment compared to the other mice. Notably, the tumour in mouse 3 did not increase in volume by calliper measurements across the experimental timeframe, although all imaging platforms detected increased growth (Additional file 1: Fig. S4B–E). Post-mortem this tumour was growing flatter and more invasively into the muscle, making it harder to detect with callipers. As a possible consequence, tumour growth was not significantly changed over days 18–22 with callipers, while via iRFP analysis in the LPT (Mathematica) was significant over days 17–21 (Fig. 1d).

### Detection of iRFP in deep tissues

**Fig. 3:**
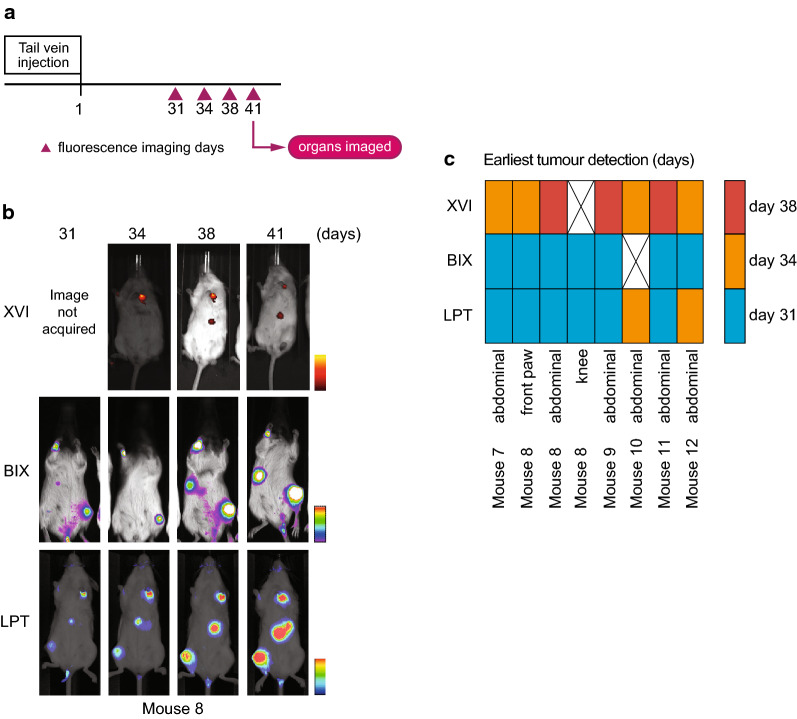
**a** Schematic representation of imaging days after tail vein injections. Organs were imaged independently on the last day. **b** Comparison of images of mouse 8 taken on each imaging day per platform. BIX mice are positioned resting on their abdomen on a glass surface with the picture taken from underneath, while in both other platforms, the mice are positioned on their backs with the image taken from above. Colour bars reflect highest and lowest values from top to bottom. **c** Heatmap depicting the earliest day that tumours were visible. Crosses are used to represent lesions which were not picked up by that platform by the end of the experiment

For subcutaneous tumours, imaging was started when tumours were palpable, precluding determination of differences in early sensitivity between imaging platforms. To determine how each platform performed on tumours that were not palpable, iRFP-expressing H1299 cells were injected in the tail vein of 6 mice, numbered mouse 7 to mouse 12, and allowed to form tumours. H1299 cells were expected to settle and grow in liver, lung and bone as previously reported [24, 25]. iRFP expression was measured on the 3 platforms on 31, 34, 38 and 41 days after injection (Fig. 3a) (similarly to the subcutaneous tumours, iRFP filters for the XVI were wrongly chosen on day one thus images were not usable). Mice were positioned in a ventral position in respect to the detector. Images of mouse 8 in all platforms and all days are depicted in Fig. 3b and images of all 6 mice in each platform on day 41 are shown in Additional file 1: Fig. S5. Mice were sacrificed after imaging on day 41 and post-mortem investigations were carried out to determine total tumour burden. Identified lesions are reported in Additional file 2: Table S4 and bright-field images are shown in Additional file 1: Fig. S5B.

As expected, during imaging abdominal tumours were detected in most mice (Additional file 1: Fig. S5A) and two limb metastases were detected in mouse 8 (Fig. 3b). Whole body imaging did not detect any metastases in the chest in the timespan of our experiment. To visualise the earliest measurement day on which each platform detected a certain signal, we displayed this as a heat map in Fig. 3c. Notably, this figure does not show all signals. We have shown images from all mice on the final day of imaging in Additional file 1: Fig. S5A. This shows the most comprehensive cohort of tumours. The only signal not apparent on day 41 is the abdominal signal of mouse 10. The LPT detected most signals across all mice, although the BIX detected the fluorescent signal from the abdomen of mouse 12 one timepoint earlier than the LPT. The BIX detected most signals on day 31 but did not detect the abdominal tumour signal of mouse 10 on any of the 4 days. The XVI detected each tumour later than the other two systems. Though this is in part due to the missed measuring day, it missed 3 of the tumours on day 34 (mouse 8 abdominal, mouse 9 abdominal and mouse 11 abdominal), which were detected by the other platforms. It was also not able to detect the knee tumour seen in mouse 8, which was seen by other platforms more than 1 week earlier (Fig. 3c).

**Fig. 4:**
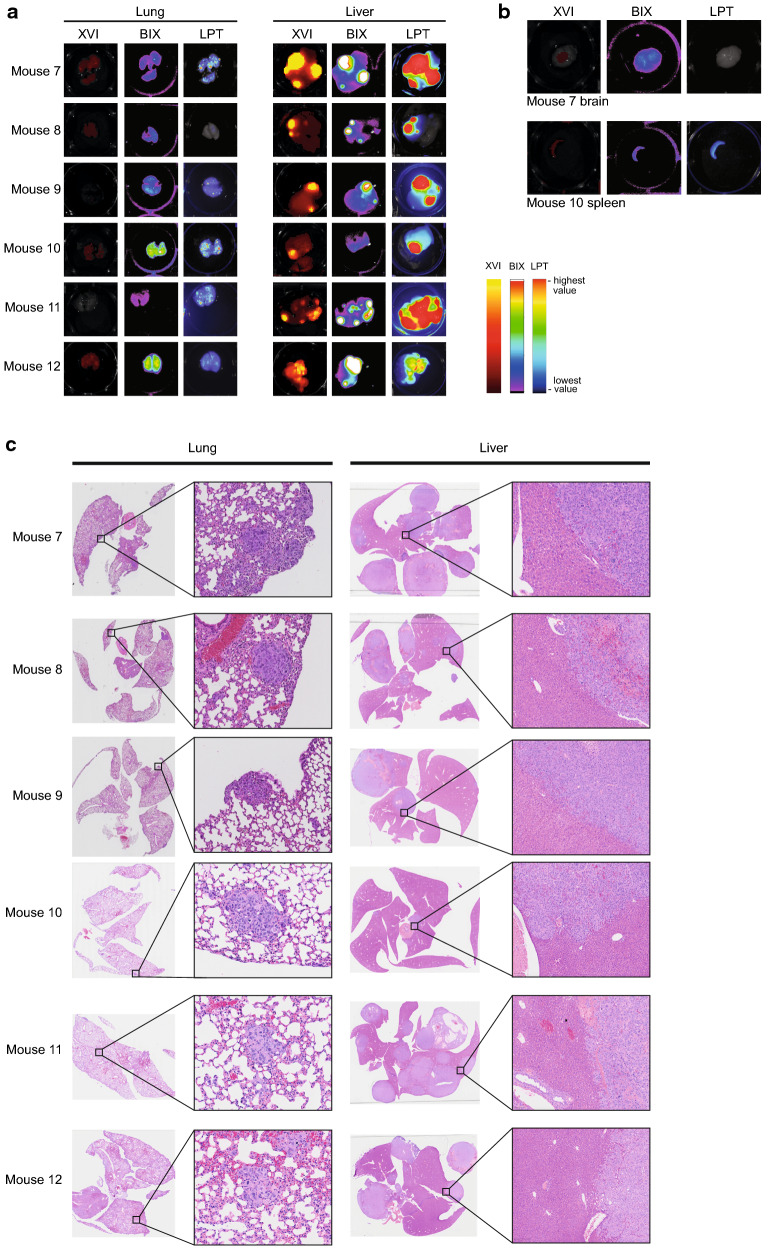
**a** Ex-vivo images of the lungs and livers for all mice in each platform. Colours are false colours as applied by platform specific software with the scales indicating highest and lowest values on the right. **b** Brain of mouse 7 and spleen of mouse 10 imaged by each platform. **c** Histological H&E sections of lungs and livers are shown as whole organs (left) or a zoom of one the tumours (right) for each mouse

**Fig. 5:**
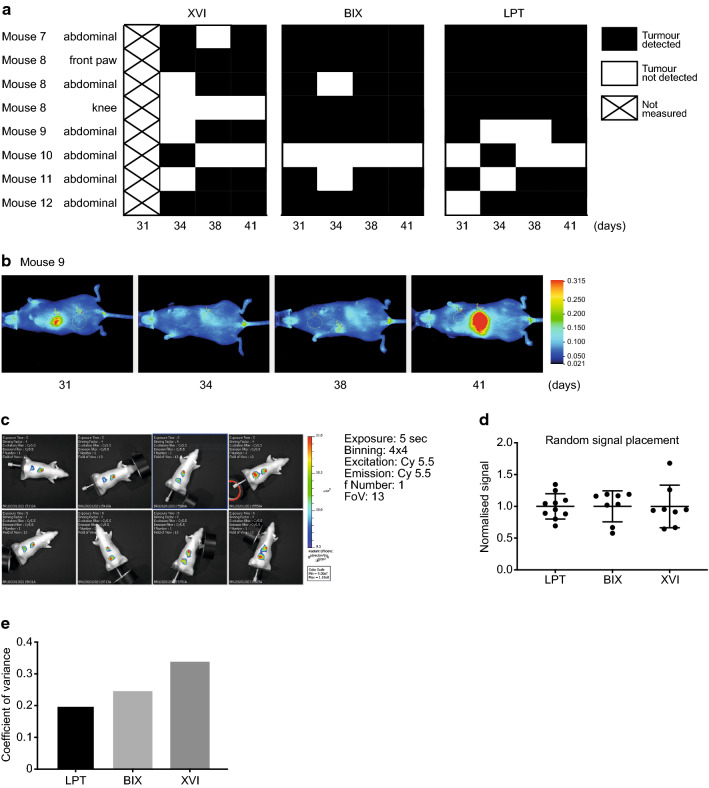
**a** Binary heatmap showing whether a tumour was or was not detected on a given day by each platform. Notably, lesions in the abdomen are scored as one lesion although in some mice more than one signal was noted on certain days. Annotation: *mx-position*, where x is the mouse number and position is a rough anatomical position seen in whole body imaging. **b** LPT images showing the disappearance and re-appearance of the abdominal tumour of mouse 9. All images are gated to day 31. **c** Example of positioning of the phantom mouse in the BIX. White text is artefactual from software and may be disregarded. **d** Variation in signal of the phantom mouse iRFP, seen in the three imaging platforms, normalised to 1. **e** coefficient of variance of phantom mouse iRFP in each platform

We next harvested livers, lung and any additional tumours found post mortem (Fig. 4a; Additional file 1: Fig. S5B). We imaged these organs after dissection, which allowed us to measure each platform’s ability to resolve very small tumours and better detect the location of the tumours detected in whole body imaging. Fluorescence was readily detected in the livers of all mice on all platforms and brightest in mouse 7 and 11. Interestingly, fluorescence in the whole-body imaging abdomen of mice 7, 8 and 12 was most pronounced (Additional file 1: Fig. S5A). The liver tumours of mouse 10 were only detected using the LPT and the XVI on day 34 in the whole-body imaging (Figs. 4a, 5a; Additional file 1: Fig. S5A). Ex vivo, the signal of this tumour was a bit more localised to one area of the liver (Fig. 4a). Histology also suggested that this liver had less large tumour areas than most of the other livers (Fig. 4c).

Our analyses of whole-body images also revealed that none of the platforms could detect lung tumours, although post mortem, lung puncta were visible in excised lungs of all mice (Fig. 4a, c; Additional file 1: Fig. S5B). Fluorescent puncta were most clearly observed with the LPT, which was able to image a large number of distinct foci in each harvested lung, whereas both the BIX and XVI only showed an indistinct mass of fluorescence across the organ. The XVI did not detect lung signals in 2 of the 6 mice (mouse 9 and 11). The LPT and the BIX were unable to detect lung tumours one mouse, mouse 8 and mouse 11 respectively, though there were tumours found in those lungs as can be seen in histology (Fig. 4c). Bone tumours in the paw and knee of mouse 8 (Fig. 3b) were also confirmed after dissection and imaging of the individual affected limbs (Additional file 1: Figs. S5B, S6A).

During imaging of tumours in each organ, a different background fluorescence was noted on each platform, which might be caused by slight differences in filter sets of each platform (Additional file 2: Table S1). In particular, the XVI and BIX both showed a very homogenous fluorescence across the whole brain, but the LPT did not (Fig. 4b). The XVI additionally detects high fluorescent background in all organs, that is less prominent in either the LPT or the BIX. The spleen had a higher background fluorescence than other organs in all platforms. In practice, this means that the LPT might be more suited to detect brain tumours, but that for all tumours background measurements for individual organs must be considered when quantifying tumours in that organ.

In order to quantify how well each machine performed to detect tumours, we calculated the percentage of detected tumours in whole body scans (Additional file 2: Table S4) compared to the actual number of tumours detected ex vivo in the individual organs (Fig. 4a, c Additional file 1: Fig. S5A, Additional file 2: Table S4). In total, ex vivo*,* we detected tumours in all 6 livers, all 6 lungs and 2 tumours in limbs. Although different nodules were detected in the liver, abdominal tumours were scored as one tumour (Fig. 4c). We then determined how often each machine was able to find these tumours in the ‘whole body’ images and calculated the percentage of detected tumours (Additional file 2: Table S4). Not accounting for the images taken on day 31, these data suggests that the XVI is the least sensitive platform for detecting deep fluorescent signals, while the BIX and LPT performed at similar levels with the LPT detecting marginally more tumours on day 34.

Once a tumour was detected, it was not always possible to re-detect it on subsequent days. To evaluate detection dynamics, we graphed the presence or absence of tumours in each platform using a binary heat map (Fig. 5a). Missing tumours in this graph could be the result of a very low signal, which if close enough to background, could appear and disappear between imaging sessions. Alternatively, positioning of the mice could be of importance to detect some tumours. Figure 5b shows an example in the LPT, where the front paw of the animal is slightly more upwards on days 31 and 41, but downwards on the intermittent measuring days. As images were taken blindly, we only noticed this subtle difference upon analyses. As the signal on day 31 is well above background, positioning seems to affect tumour detection. We therefore wanted to determine how much variation we could create by changing the position of a phantom mouse with a signal close to the iRFP spectrum (Alexa Fluor 680) (Fig. 5c) and how important the maximum variation would be in each machine. The phantom mouse, used to calibrate machines, is a plastic mouse shaped object with cavities at various depths into which fluorescence-coated probes can be inserted to check how well a mouse can be imaged with this probe. The signal was normalised to 1 to be able to display the variation of each platform next to each other (Fig. 5d) and the coefficient of variance was calculated (Fig. 5e). These results indicate that the highest variation was seen in the XVI with 35% variation and the lowest in the LPT 20%. Positioning differences are therefore a limiting factor for the accurate detection of small visceral tumours, making quantification of such deep tissue tumours difficult and highly variable.

## Discussion

We evaluated the performance of different iRFP imaging platforms to quantify growth of subcutaneous tumours and detect deep-tissue tumours arising from injected iRFP expressing cells. Comparing three platforms, the Xenogen VivoVision IVIS 200 (XVI), the Bruker In-Vivo Xtreme (BIX) and the Li-Cor Pearl Trilogy (LPT), it is clear that all three are able to measure iRFP expressed in tumours, but that each individual platform has features which might suit certain experiments better than others (Additional file 2: Table S1).

The level of fluorescence in a cell or tissue is dependent on a variety of factors including gene copy number, the activity of the promotor and protein stability, which all lead to differences in fluorescence that affect measurements. To rule out such variations, we compared tumours established from cells expressing high and stable levels of iRFP. Here we used iRFP713, the most frequently used construct in near-infrared imaging. Many studies have used the IVIS imaging systems to study deep-tissue tumours [10, 17–20], a smaller number have used the LPT [11, 14] and others have used custom-built machines [15, 21]. The BIX imaging system has been used to study iRFP expression in mice, for example to study adipose tissue [26], but we found no reports on its use for tumour growth or metastasis using iRFP.

The most frequently used method for subcutaneous tumour measurements is using callipers. Previous comparisons between GFP fluorescence and tumour volume showed a good correlation between these parameters [27, 28]. We also observed that careful, objective iRFP measurements showed variation similar to calliper measurements. However, one tumour was not picked up as growing using calliper (mouse 3), but was detected growing in all imaging platforms. This tumour was flatter and invasive into the muscle, something that can happen dependent on the injection and the cell type one investigates and points towards an advantage for fluorescent imaging. While the callipers estimated a 1.5-fold increase in tumour volume, the imaging platforms with proprietary software generally measured between a 3.6- and 4.1-fold increase in fluorescence intensity as a surrogate of tumour volume. This seemed mainly due to an overestimation of intensity in the proprietary software possibly due to user misjudgements in subjective gating, as analyses of raw images with 3D-guided gating using our Mathematica applet showed a 1.7–2.4-fold increase in tumour growth over the same days. In an experimental setting, proprietary software analysis could therefore lead to an overestimation of the actual differences between experimental conditions.

Another consideration is that images in the XVI reached the maximum intensity at the last day and the BIX was close to reaching this maximum limit. Exposure time, aperture or binning can be changed to avoid image saturation, but this would lead to difficulties in comparing the intensities between days. Determining constant settings ahead of time is difficult, especially when the total dynamic range of a given platform is low. Although theoretically this can be compensated for as fluorescent emission is linear in time, in practice day-to-day variation such as feeding state or urine volumes will lead to variability in measurements. According to marketing material, and confirmed experimentally, the LPT has an advantage of a larger dynamic range by internally combining several exposures, and is therefore able to image at the same settings throughout an experiment without saturating.

One of the biggest challenges in determining growth of tumours in vivo, is setting the intensity thresholds (gating) to help define tumour boundaries. For example, images can be gated to show signal in the smallest tumour on the first day of imaging, with settings maintained to gate each subsequent image. Alternatively, images can be gated on the last day to show the grown tumours, with the same settings applied to each preceding day. This can result in drastic differences in the visualisation of the tumours, and therefore in defining their boundaries and determining their signal intensity. Regardless of the method chosen, human error persists as the optimum tumour boundaries have to be chosen either by eye or automatically (automatic detection was very inconsistent). Each of the manufacturer’s software removes background signal when gating images, making assessment of where the actual signal starts challenging. By analysing the raw TIF files in 3D using our applet developed in Mathematica, we found it easier to determine tumour boundaries when able to simultaneously view background signals, meaning that the process of gating the images is not required. This suggests that improvements made in the software of each of these platforms could lead to more accurate results and less bias by the researcher in gating for subsequent quantification.

Previous experiments by Lai et al. [20] showed that iRFP was more reliable and more sensitive to detect early changes in tumour growth than calliper measurements. Our experimental setup for the subcutaneous tumours did not allow strong conclusions to be drawn over the sensitivity of iRFP measurements in subcutaneous growth between the 3 platforms. However, we were able to show that the BIX and LPT platforms were able to image liver and bone tumours early and reliably from tail vein injected H1299 cells. H1299 administered in the blood of mice have previously been shown to settle in the liver, brain, ovaries, bone and most predominantly in the lung [25]. Remarkably, lung tumours were not detected in whole body scans in our study, despite the fact that we could detect them ex vivo in these organs. The chest wall forms a barrier for detecting fluorescent signals, but Lai et al. previously reported iRFP signals from lung tumours using whole body imaging [20]. However, their work also reveals that iRFP signal is quenched by the chest wall and reduced with the depth of tumours, suggesting that the tumours we detected post mortem in the lung were too small to be detected in the whole-body scan. We did detect liver and bone tumours, but we did not detect any brain tumours in either the whole-body scans, the ex vivo images, or by post mortem examination.

We found that some imaging platforms were unable to detect certain tumours. This is important in any experiments in which missing a lesion could be crucial to the outcome. Differences between machines could be due to the detector being positioned above the imaging bed or below. In the LPT and the XVI, the detector is positioned above the mice, whereas the detector of the BIX it is positioned below the imaging bed, resulting in compression of the skin and organs, potentially reducing scattering and absorption of light with the consequence of some tumours being undetected. The BIX imaging arrangement, however, may also provide a level of consistency which the other platforms may be missing. Because the animal is flattened against the glass, this allows for a greater consistency of limb placement than in the LPT or BIX when not using tape to secure. Surprisingly, despite the more fixed body position, the BIX did not detect more tumours than the LPT and missed tumours as often as the LPT, reinforcing the idea that the LPT was more sensitive in picking up tumours. The mixed effects of organ movement and limb placement are hard to untangle, but it is clear that both cause variation in detecting deep tissue iRFP signals.

Despite the fact that we could not capture images on day 14 of the XVI, our results suggest that both the LPT and the BIX were more sensitive in picking up deep tissue tumours earlier than the XVI. LPT marketing indicates that low background signal is achieved through a monochromatic near-infrared laser, which might give the LPT a detection advantage. In addition, the XVI seemed to have higher background levels during ex vivo imaging compared to the other platforms. We used higher binning settings in the XVI that were recommended by other users to enable earlier detection of tumours, albeit at the loss of resolution. The individual imaging of organs has shown that even with a high binning of 8 × 8, the XVI has enough resolution to detect smaller lesions in the liver (e.g. mouse 12 compared to the other platforms, Fig. 4a), suggesting that this high binning did not underlie the lack of detecting tumours. Notably, however, because each mouse had a slightly different pattern of tumours, these mice cannot be counted as replicates, and we can therefore not draw conclusions about the general reliability of the platforms in these settings. Instead, we have a real-world scenario in which each mouse is imaged only once at each timepoint and many slightly different mice. The results of missing tumours can therefore be seen as an example of data which may have been missed during a routine study, and a sufficient reason to ensure that multiple images were taken for each animal at each time point. Additionally, we only imaged our animals in a supine position. It is conceivable that other tumours may have been detected if the mice were imaged in different positions.

Each platform displayed strengths and weaknesses throughout our testing. All platforms are well suited to measure subcutaneous growth, but the LPT had the highest dynamic range in our dilution series tests. More importantly, when imaging organs and deep tissue tumours the BIX and the LPT performed slightly better in detecting more tumours and the resolution of the LPT allowed to see more details of small tumours in lungs post mortem. It is worrying that mice had many small lung tumours that were not seen in any of the platforms in the whole-body scans. The question is whether other techniques would have picked these up and whether MRI or luciferase are better for detecting deep-tissue tumours earlier.

Our results as a whole conclude that iRFP imaging, growing in popularity, is a good non-invasive method for tracking tumour growth, though it also serves as a warning that care must be taken when using this method. Our findings show that different platforms can give different answers about the magnitude of growth of subcutaneous tumours, and can also detect deeper tumours differently from one another. Researchers should consider different solutions for obtaining information from raw images as taken from the platforms. Our results using Mousetensity, the Mathematica package which we developed show that we were able to extract more comparable data between platforms, and use our own approach to image segmentation which we found to be more reliable. As the use of this technology continues to expand, we hope that researchers will take these findings on board, leading to more reliable results.

## Methods

### Stable cell line creation

iRFP 713 was a kind gift from Drs A. Hock and K Vousden. H1299 and A431 cells were obtained from ATCC (www.ATCC.org), authenticated using STR profiling and checked against the ATCC reference and screened against mycoplasm. Cells were grown in DMEM (Gibco) supplemented with FBS (Sigma) at 37° and transfected with iRFP713 (Ex688, Em713) using lipofectamine 2000 (ThermoFisher Scientific) according to the manufacturer’s instruction. iRFP in cells is then expressed under a CMV promotor and stable cells were generated by growing cells in 96-well plates in serial dilutions and identifying positive clones using the Li-Cor Odyssey Sa platform. Clones with the highest expression levels were selected for further studies.

### Creation of imaging test plate

A confluent T75 flask of iRFP-expressing A431 cells was trypsinised (ThermoFisher Scientific), pelleted and fixed with 4% paraformaldehyde in PBS (10 min). The fixed cells were resuspended in 1.5 ml of pre-warmed 0.5% agarose (Invitrogen). Similarly, 5 µl of IRDye 680RD antibodies (Li-Cor) were diluted into 1.5 ml of agarose. The resulting solutions were then used to create tenfold serial dilutions. 450 µl of each solution was used to fill triplicate wells in a black 96-well CellCarrier plate (PerkinElmer) and allow to solidify at RT. Wells were over-filled to form convex menisci which were then sliced off to create uniform flat surfaces. To allow each platform to perform optimally, the acquisition settings were changed between iRFP and 680RD acquisition (Additional file 2: Table S2). Of note, both were imaged with the same settings on the LPT, since no settings other than binning can be changed on this platform. Images were analysed separately with each platform’s image analysis software. Average and SD were calculated using GraphPad Prism v9.

### Animals and tumour formation

All procedures involving mice were carried out under the UK Home Office project licence number P449972E8. Female NOD SCID gamma mice (JAX NSG) were obtained from internal breeding colonies. Upon arrival mice were acclimatised for one week. Experiments commenced when they were between 42 and 46 days old and weighing over 20 g. All mice appeared in good health and were randomly allocated to the groups by an independent technician not involved in our study. Mice were kept in groups of 3 per cage (tecniplast Blue-line IVC) with food (irradiated RM-3, SDS), environmental enrichment (nesting material, cardboard tunnels, wooden chew sticks, hemp nestlets and handling tunnels) and water (filtered 0.2 micron, bottle change 2 × per week) ad libitum with Datesand Fibreton 6 as bedding. Weight was recorded twice per week. Tumour burden was monitored twice per week, until palpable, then calliper-measured twice per week. Tumour volume was estimated by using the formula (length × width^2^)/2. 12 mice were used overall, with 6 receiving injections leading to subcutaneous tumours, and 6 receiving tail-vein injections to lead to a metastasis-like model. Previous calculations using imaging mCherry had shown that 6 mice were sufficient to show differences in xenograft growth using in vivo imaging in a relevant cancer setting [29]. For subcutaneous and tail vein injections, cells were tested for mycoplasma prior to the procedure. A total of 2 × 10^6^ H1299 cells in 100 μl 50% geltrex/PBS (Gibco) were injected subcutaneously into the left flank of another 6 mice (1–6). A total of 2 × 10^6^ H1299 cells in 100 μl PBS were injected into the tail vein of 6 mice (7–12). Calliper measurements were performed independently by blinded mouse husbandry specialists. The timeline of callipers and imaging was set before the experiment commenced and due to availability in the imaging room unable to be performed on the same day. Calliper measurements were done prior to imaging the following day. NSG Mice were chosen because they are a commonly used model to study the growth and spread of xenograft tumours. No animals were excluded from this study.

### Determination of intra-platform signal variation

To determine the level of signal variation found within each platform, we used the XFM-2 phantom mouse carrying an Alexa Fluor 680 emitter (Caliper LifeSciences). The phantom was repeatedly imaged with each platform to introduce variation. The phantom was placed randomly, repeatedly into different positions by varying the height and rotation of the phantom, as well significantly changing the position and rotation of the emitter (180° rotations, and up to 5 mm changes in depth) in order to maximise variations.

### In vivo imaging of iRFP

Prior to imaging, mouse anaesthesia was induced using 4 L/min of oxygenated Isoflurane (Merial Animal Health, 50,878) and maintained (2 L/min) in the imaging platforms (37 °C) using the platform-specific nose cones. For the Xenogen VivoVision IVIS 200 (XVI) and the Bruker in-Vivo Xtreme (BIX), anaesthetised mice were placed in groups of 3 and for the Li-Cor Pearl Trilogy (LPT) individually, according to their cage number, into the chamber and aligned with the nose cones, or in the case of the XVI, the laser guided field of view. Mice with subcutaneous tumours were placed on their side with the flank containing the tumour facing the camera. Tail vein injected mice were placed dorsally in the XVI and LPT and ventrally in the BIX, in order for the abdomen to face the camera. Brightfield and fluorescent images were obtained with each machine. Image acquisition settings were initially based on feedback/recommendations by regular users of each platform, or from sales representatives. The LPT was set to a “medium” binning (2 × 2), the XVI was set to “medium” binning (binning of 8 × 8) and the BIX acquired images without binning Imaging operators were blinded by ensuring that they had not looked at the previous days’ image before taking the current image. Separate operators placed the mouse, and took the image so that only the former needed to know which mouse was being imaged. Imaging was performed in a 2-week period when all platforms were available, for fairest comparison. We set imaging frequency at twice per week to allow mice time to recover from anaesthesia.

### Image analysis

In line with best practice, backgrounds were first defined on each mouse using an ellipse tool, selecting a region similar in size to the tumour but in an area with only background signal at the shoulder contralateral to the visibly positive signals. The threshold for visualisation was set individually for each mouse so that the tumour was visible on the first day of measurements. This threshold was applied to each image of this mouse across each day. This process was repeated for each mouse. The tumour regions were then defined using the freehand tool, as the available automatic selection tools were unreliable and variable. Values were then extracted from the software and analysed using GraphPad Prism v9. To calculate tumour intensities from each mouse independently, we created a Wolfram Mathematica applet [Wolfram Research, Inc., Mathematica (Version 12.1, Champaign, IL (2020))], to make 3D models of the signal and to calculate tumour intensities.

3D interpretations of the infrared data were generated from TIF files generated by each platform. Raw TIFs were first cropped to show only the tumour and a small area around it, taking care to ensure that the cropped area was approximately the same size each time. The TIFs were then loaded into the Mathematica Applet (MouseTensity) where the pixel values were normalised to lie within the range 0 to 1. A rotatable 3D plot was then generated by plotting the (normalised) intensity as the Z-axis, which enabled the visualisation of the entire mouse, including clear definition of the fur, as the background intensity is related to the distance from the detector. This allowed us to create a plane through the 3D plot, in a similar way to that which is done in the proprietary software for all three imaging platforms, but with the benefit of an easier visualisation of the surrounding area. This plane can then be moved vertically until the plane transcribes the 3D object in a shape defining the boundaries of the signal produced by the tumour, separating it from the rest of the body of the mouse. An initial value for this plane is provided via a thresholding method based on enthalpy, which could then be adjusted by the user [30]. This cut-off is then used to define the area on the TIF which is considered to be the signal, and then all measurements are taken directly from the raw, unscaled, image. The height of the plane on the z-axis can be used to determine the level of signal above the background, which can then be subtracted. This applet is deposited on: https://zenodo.org/record/4335047.

### Histology

Livers and lungs were fixed in formalin, cut and stained for H&E. Slides were scanned in the Olympus VS200.

### Statistics

GraphPad Prism 9 was used make graphs and to calculate statistical differences using a one-way ANOVA as described in the figure legends. P-values of less than 0.05 were considered statistically significant.

## Supplementary Information

**Additional file 1** **Figure S1** (A) Normalised signal of a logarithmic dilution of fixed iRFP expressing A431 cells suspended in low-melt agarose measured at two different times on the LPT: Before all other machines (first reading), and after all other machines (final reading). (B) Tumour signal or background signal per pixel per mouse on each day, measured on the LPT. **Figure S2** (A-C) Tumour intensity images generated using our Mathematica MouseTensity software. Subcutaneous tumours from all 6 mice were imaged using all 3 platforms (A)XVI, (B)BIX, (C) LPT on day 17. The units for the x- and y-axes are pixels, and standardised units for the z-axis. **Figure S3** (A-C) Tumour intensity images generated using our Mathematica MouseTensity software. Subcutaneous tumours from all 6 mice were imaged using all 3 platforms (A) XVI, (B) BIX, (C) LPT on day 24. The units for the x- and y-axes are pixels, and standardised units for the z-axis. **Figure S4** (A) Example of saturated pixels detected in the XVI (Mouse 1 and 4). Only the saturated pixels are shown on the right and the tumour is shown on the left. Mouse 4, day 21 is representative of a low level of saturation, but this would still affect final quantification, while mouse 1 day 24 is representative of an obvious overexposure (B-D). Line graphs of the growth of subcutaneous tumours to track tumour growth in each mouse. These are the same data as Fig. 1E-G, but are now shown as lines, coloured per mouse. (E) Calliper measurements coloured to show growth of tumours over time. These are the same data as Fig. 1B, but are now shown as lines, coloured per mouse. **Figure S5**. (A) Examples of full body images of all mice on the last day of imaging on each platform. (B) Brightfield images of excised organs, taken in the LPT. Liver and lungs are shown for all mice, and front paw and knee that clearly showed a signal is shown for mouse 8. **Figure S6**. Fluorescent images of the knee and front paw of mouse 8 in all imaging platforms.

**Additional file 2**. **Table S1** Details of the features of each tested platform, obtained from public data. Some information has been independently calculated/determined by the authors. **Table S2** Image acquisition parameters for each platform for the plate, phantom and mice. Information in quotations is naming provided by software. **Table S3** Statistics from Figure 1 EFG. Full statistical output from ANOVA, with Tukey comparisons. **Table S4** Counts of tumours arising from tail vein injections, including percentages of the total number of tumours detected by each platform. The total tumour counts are listed in a separate table.

## Data Availability

Raw image data, Software package “Mousetensity” as a Mathematica package and the dataset(s) supporting the conclusions of this article are available in the zenodo repository, 10.5281/zenodo.4335047. All other raw data is available upon request.

## Abbreviations

BIX: Bruker In-Vivo Xtreme
XVI: Xenogen VivoVision IVIS 200
LPT: Li-Cor Pearl Trilogy
iRFP: Near-infrared fluorescent protein
RT: Room temperature
GFP: Green fluorescent protein
MRI: Magnetic resonance imaging
CCD: Charge-coupled device
NSG: NOD scid gamma

## References

[1] Euhus DM, Hudd C, LaRegina MC, Johnson FE (1986). Tumor measurement in the nude mouse. J Surg Oncol.

[2] Tomayko MM, Reynolds CP (1989). Determination of subcutaneous tumor size in athymic (nude) mice. Cancer Chemother Pharmacol.

[3] Wang Y, Tseng JC, Sun Y, Beck AH, Kung AL (2015). Noninvasive imaging of tumor burden and molecular pathways in mouse models of cancer. Cold Spring Harb Protoc.

[4] Burgos JS (2003). Time course of bioluminescent signal in orthotopic and heterotopic brain tumors in nude mice. Biotechniques.

[5] Khalil AA (2013). Subcutaneous administration of d-luciferin is an effective alternative to intraperitoneal injection in bioluminescence imaging of xenograft tumors in nude mice. ISRN Mol Imag.

[6] Shaner NC (2004). Improved monomeric red, orange and yellow fluorescent proteins derived from Discosoma sp. red fluorescent protein. Nat Biotechnol.

[7] Bhaumik S, DePuy J, Klimash J (2007). Strategies to minimize background autofluorescence in live mice during noninvasive fluorescence optical imaging. Lab Anim.

[8] Zhang X, Bloch S, Akers W, Achilefu S. Near-infrared molecular probes for in vivo imaging. In: Current protocols in cytometry Chapter 12, Unit 12.27; 2012. 10.1002/0471142956.cy1227s60 PMC3334312 22470154

[9] Filonov GS (2011). Bright and stable near-infrared fluorescent protein for in vivo imaging. Nat Biotechnol.

[10] Filonov GS (2012). Deep-tissue photoacoustic tomography of a genetically encoded near-infrared fluorescent probe. Angewandte Chemie (Intern ed Eng).

[11] Hock AK (2017). Development of an inducible mouse model of iRFP713 to track recombinase activity and tumour development in vivo. Sci Rep.

[12] Hock AK (2014). iRFP is a sensitive marker for cell number and tumor growth in high-throughput systems. Cell Cycle.

[13] Genevois C, Loiseau H, Couillaud F (2016). In vivo follow-up of brain tumor growth via bioluminescence imaging and fluorescence tomography. Intern J Mol Sci.

[14] Jiguet-Jiglaire C (2014). Noninvasive near-infrared fluorescent protein-based imaging of tumor progression and metastases in deep organs and intraosseous tissues. J Biomed Opt.

[15] Lu Y (2013). In vivo imaging of orthotopic prostate cancer with far-red gene reporter fluorescence tomography and in vivo and ex vivo validation. J Biomed Opt.

[16] Tanaka N (2016). Application of infrared-based molecular imaging to a mouse model with head and neck cancer. Head Neck.

[17] Tran MT (2014). In vivo image analysis using iRFP transgenic mice. Exp Anim.

[18] Wilson AL (2018). Non-invasive fluorescent monitoring of ovarian cancer in an immunocompetent mouse model. Cancers.

[19] Yu D (2014). An improved monomeric infrared fluorescent protein for neuronal and tumour brain imaging. Nat Commun.

[20] Lai CW (2016). Using dual fluorescence reporting genes to establish an in vivo imaging model of orthotopic lung adenocarcinoma in mice. Mol Imag Biol.

[21] Rice WL, Shcherbakova DM, Verkhusha VV, Kumar AT (2015). In vivo tomographic imaging of deep-seated cancer using fluorescence lifetime contrast. Can Res.

[22] Luis-Ravelo D (2011). Tumor-stromal interactions of the bone microenvironment: in vitro findings and potential in vivo relevance in metastatic lung cancer models. Clin Exp Metas.

[23] Fukuda A (2019). Non-invasive in vivo imaging of UCP1 expression in live mice via near-infrared fluorescent protein iRFP720. PLoS ONE.

[24] Singla AK, Downey CM, Bebb GD, Jirik FR (2015). Characterization of a murine model of metastatic human non-small cell lung cancer and effect of CXCR4 inhibition on the growth of metastases. Oncoscience.

[25] Yang L (2015). MircoRNA-33a inhibits epithelial-to-mesenchymal transition and metastasis and could be a prognostic marker in non-small cell lung cancer. Sci Rep.

[26] Chan XHD (2018). Multimodal imaging approach to monitor browning of adipose tissue in vivo. J Lipid Res.

[27] Diehn FE (2002). Noninvasive fluorescent imaging reliably estimates biomass in vivo. Biotechniques.

[28] Choy G (2003). Comparison of noninvasive fluorescent and bioluminescent small animal optical imaging. Biotechniques.

[29] Phatak V (2021). Mutant p53 promotes RCP-dependent chemoresistance coinciding with increased delivery of P-glycoprotein to the plasma membrane. Cell Death Dis.

[30] Kapur JN, Sahoo PK, Wong AKC (1985). A new method for gray-level picture thresholding using the entropy of the histogram. Comput Vis Gr Image Process.

