# Supplementary material for: iRFP (near-infrared fluorescent protein) imaging of subcutaneous and deep tissue tumours in mice highlights differences between imaging platforms
Source: Cancer Cell Int. 2021 May 3;21:247. doi: 10.1186/s12935-021-01918-8 (PMC8091726; doi:10.1186/s12935-021-01918-8)

Figure S1

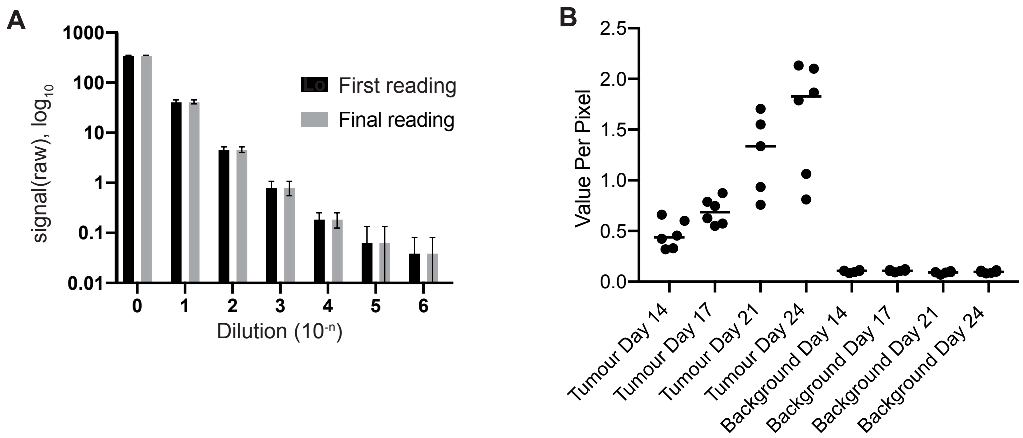
A
1000
100
10
1
0.1
0.01
signal(raw), log10
First reading
Final reading
0
1
2
3
4
5
6
Dilution (10-n)
B
2.5
2.0
1.5
1.0
0.5
0.0
Value Per Pixel
Tumour Day 14
Tumour Day 17
Tumour Day 21
Tumour Day 24
Background Day 14
Background Day 17
Background Day 21
Background Day 24

Figure S2

A Xenogen Vivovision IVIS (XVI)
Day 17

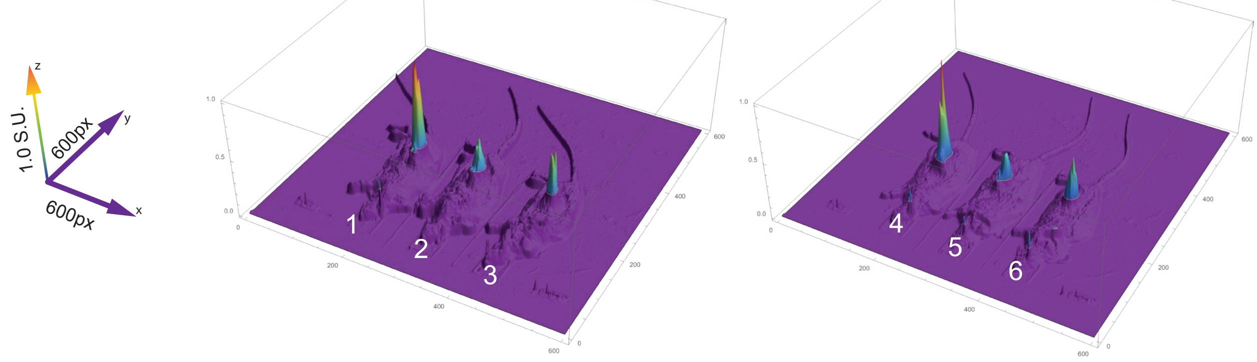

B Bruker InVivo Xtreme (BIX)
Day 17

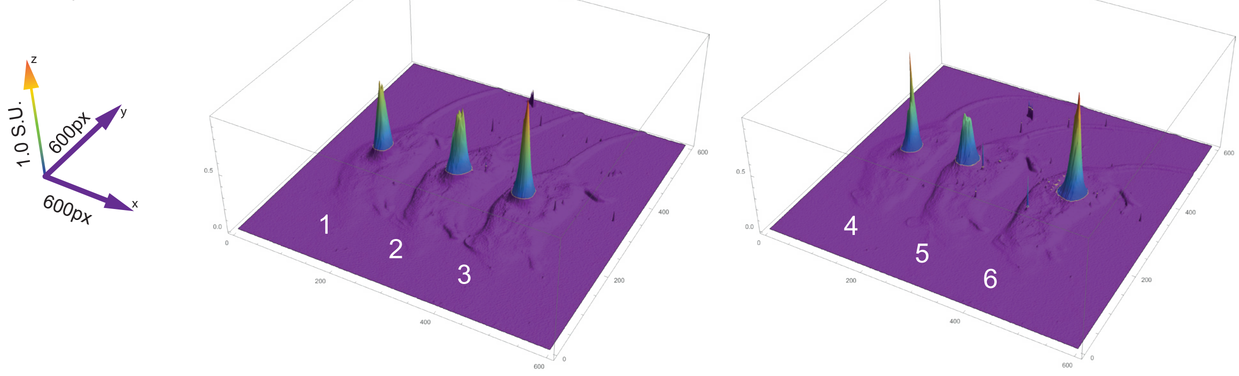

C Li-Cor Pearl Trilogy (LPT)
Day 17

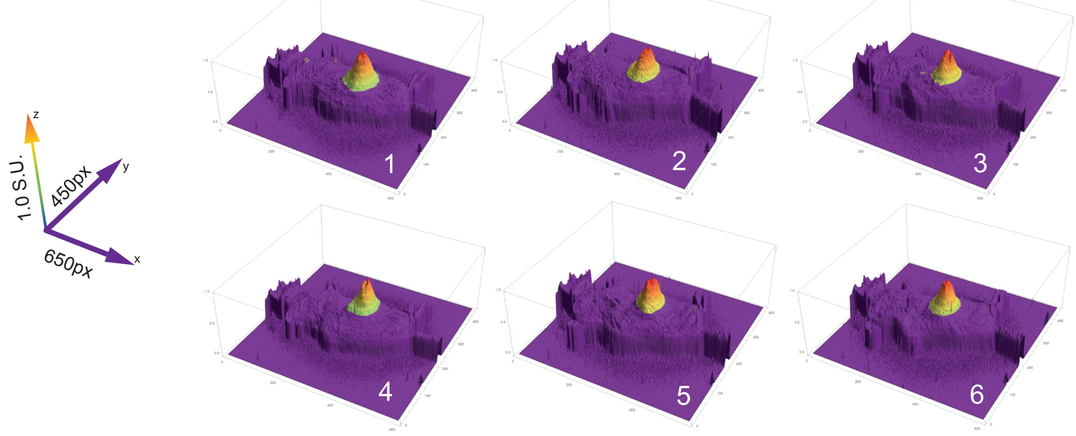

Figure S3

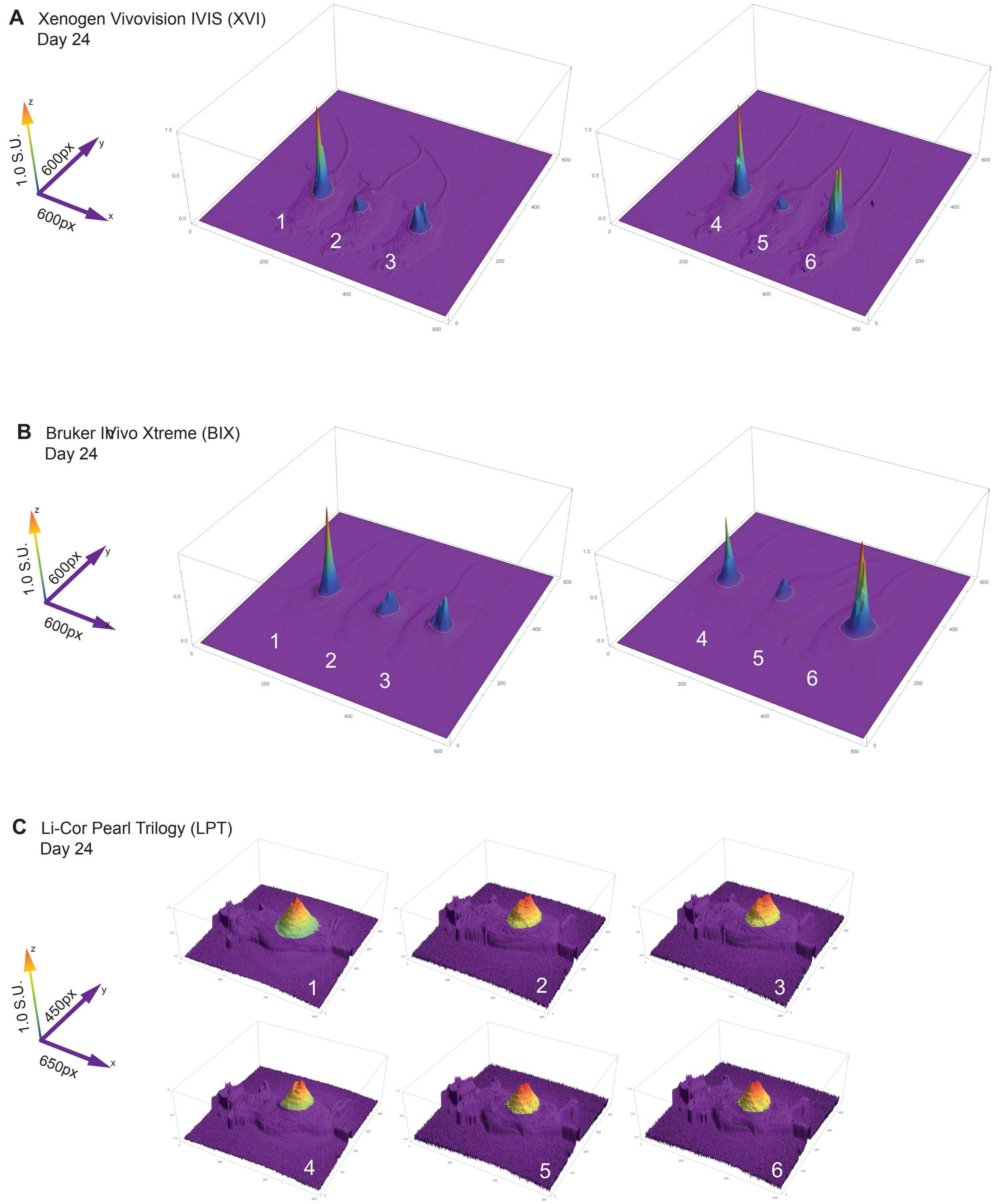
A
Xenogen Vivovision IVIS (XVI)
Day 24
z
y
x
1.0 S.U.
600px
600px
1
2
3
4
5
6
B
Bruker InVivo Xtreme (BIX)
Day 24
z
y
x
1.0 S.U.
600px
600px
1
2
3
4
5
6
C
Li-Cor Pearl Trilogy (LPT)
Day 24
z
y
x
1.0 S.U.
450px
650px
1
2
3
4
5
6

Figure S4

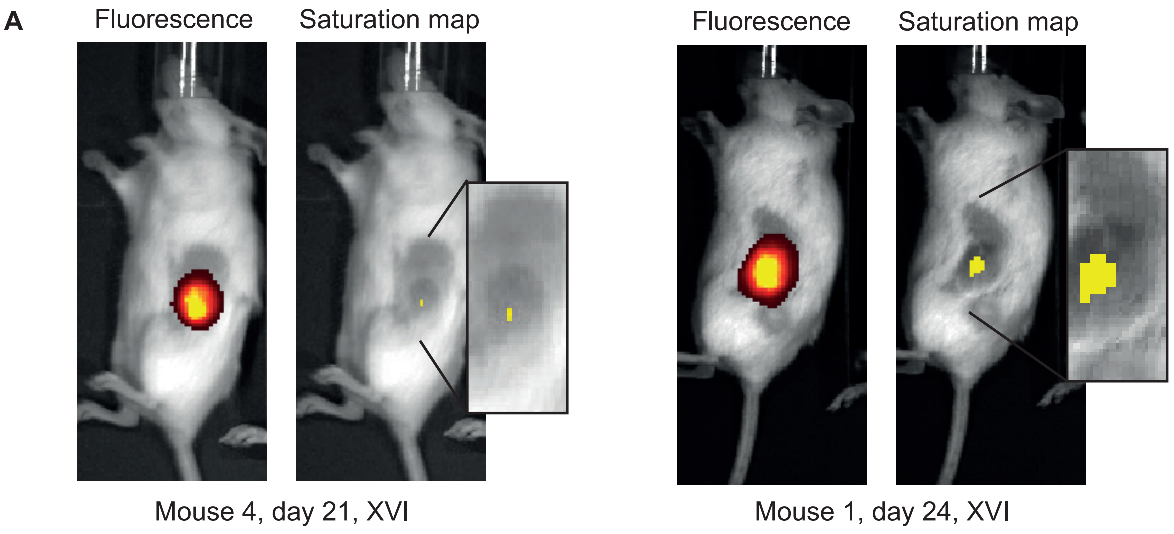

A
Fluorescence
Saturation map
Mouse 4, day 21, XVI
Fluorescence
Saturation map
Mouse 1, day 24, XVI

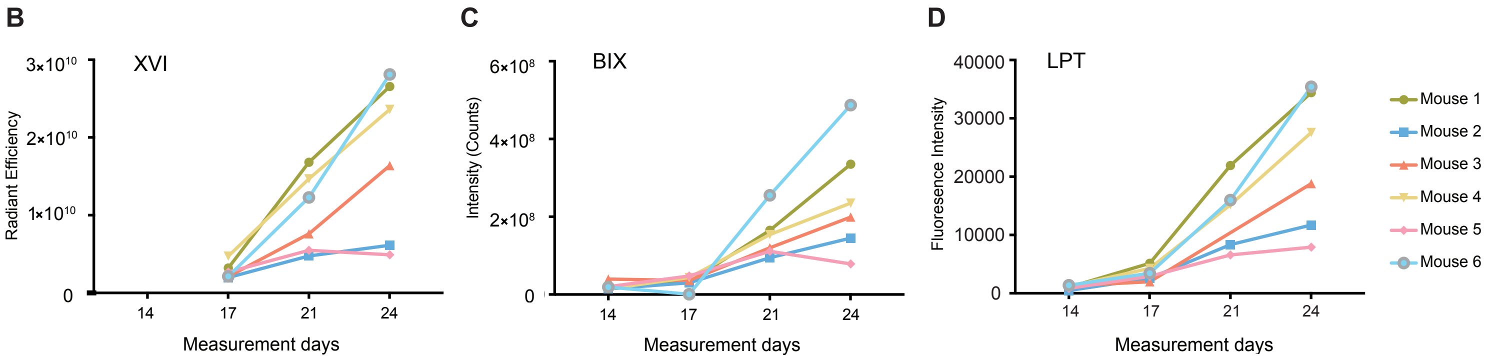

B
XVI
Radiant Efficiency
3×10¹⁰
2×10¹⁰
1×10¹⁰
0
14
17
21
24
Measurement days
C
BIX
Intensity (Counts)
6×10⁸
4×10⁸
2×10⁸
0
14
17
21
24
Measurement days
D
LPT
Fluoresence Intensity
40000
30000
20000
10000
0
14
17
21
24
Measurement days
Mouse 1
Mouse 2
Mouse 3
Mouse 4
Mouse 5
Mouse 6

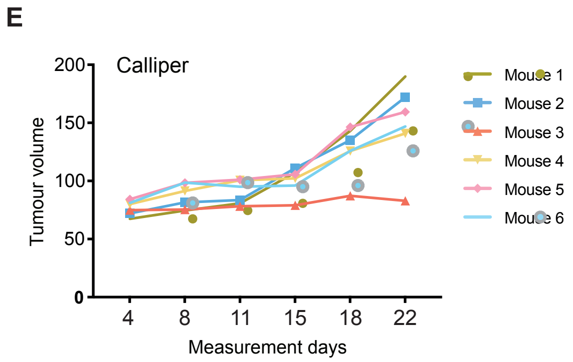

E
Calliper
Tumour volume
200
150
100
50
0
4
8
11
15
18
22
Measurement days
Mouse 1
Mouse 2
Mouse 3
Mouse 4
Mouse 5
Mouse 6

Figure S5

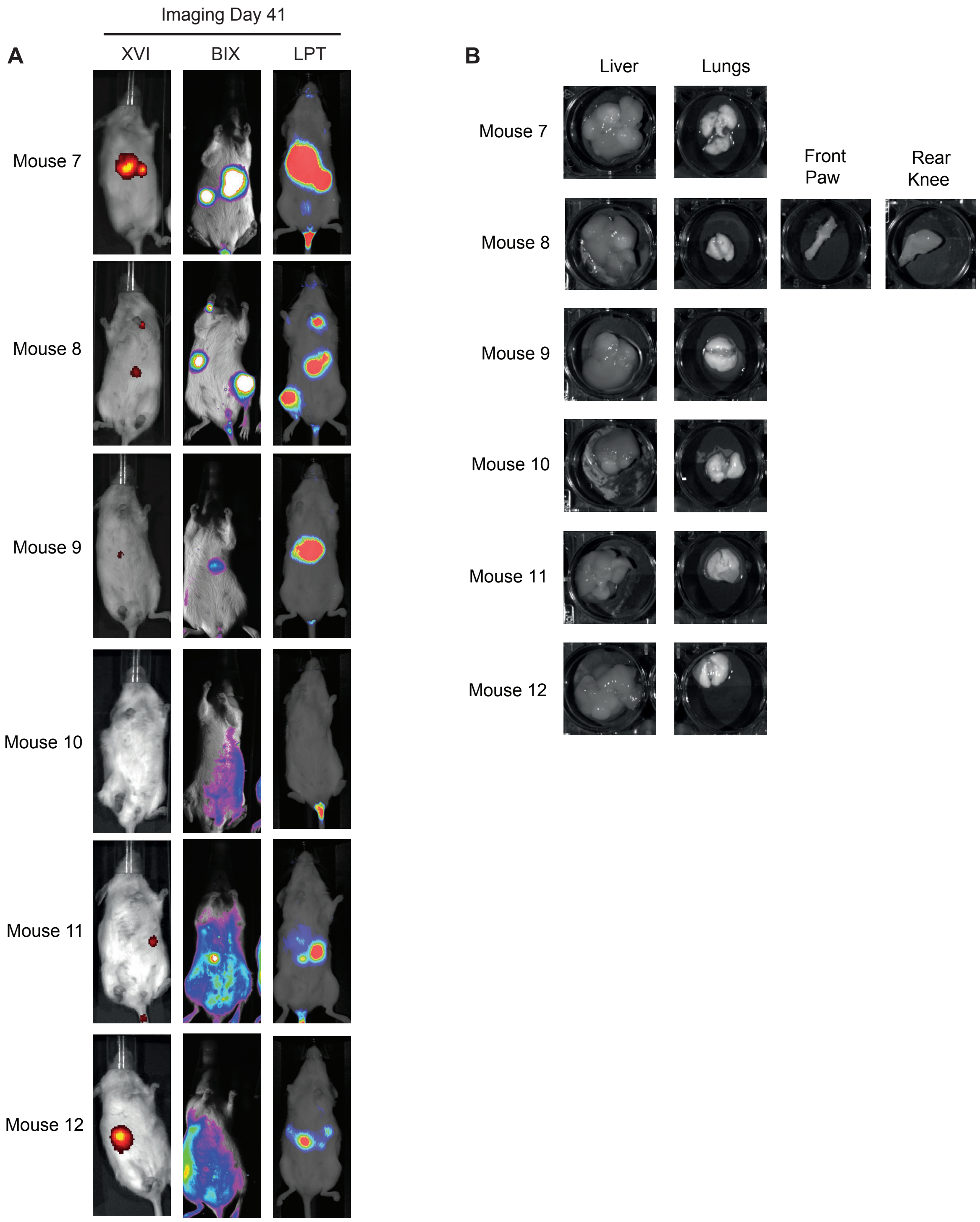
A
Imaging Day 41
XVI
BIX
LPT
Mouse 7
Mouse 8
Mouse 9
Mouse 10
Mouse 11
Mouse 12
B
Liver
Lungs
Front Paw
Rear Knee
Mouse 7
Mouse 8
Mouse 9
Mouse 10
Mouse 11
Mouse 12

Figure S6

A

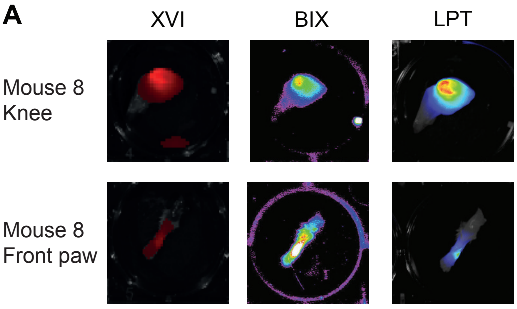

Supplement: Supplementary file 1 — Additional file 1 Figure S1 (A) Normalised signal of a logarithmic dilution of fixed iRFP expressing A431 cells suspended in low-melt agarose measured at two different times on the LPT: Before all other machines (first reading), and after all other machines (final reading). (B) Tumour signal or background signal per pixel per mouse on each day, measured on the LPT. Figure S2 (A-C) Tumour intensity images generated using our Mathematica MouseTensity software. Subcutaneous tumours from all 6 mice were imaged using all 3 platforms (A)XVI, (B)BIX, (C) LPT on day 17. The units for the x- and y-axes are pixels, and standardised units for the z-axis. Figure S3 (A-C) Tumour intensity images generated using our Mathematica MouseTensity software. Subcutaneous tumours from all 6 mice were imaged using all 3 platforms (A) XVI, (B) BIX, (C) LPT on day 24. The units for the x- and y-axes are pixels, and standardised units for the z-axis. Figure S4 (A) Example of saturated pixels detected in the XVI (Mouse 1 and 4). Only the saturated pixels are shown on the right and the tumour is shown on the left. Mouse 4, day 21 is representative of a low level of saturation, but this would still affect final quantification, while mouse 1 day 24 is representative of an obvious overexposure (B-D). Line graphs of the growth of subcutaneous tumours to track tumour growth in each mouse. These are the same data as Fig. 1E-G, but are now shown as lines, coloured per mouse. (E) Calliper measurements coloured to show growth of tumours over time. These are the same data as Fig. 1B, but are now shown as lines, coloured per mouse. Figure S5. (A) Examples of full body images of all mice on the last day of imaging on each platform. (B) Brightfield images of excised organs, taken in the LPT. Liver and lungs are shown for all mice, and front paw and knee that clearly showed a signal is shown for mouse 8. Figure S6. Fluorescent images of the knee and front paw of mouse 8 in all imaging platforms. [file 12935_2021_1918_MOESM1_ESM.pdf]
