# Supplementary material for: iRFP (near-infrared fluorescent protein) imaging of subcutaneous and deep tissue tumours in mice highlights differences between imaging platforms
Source: Cancer Cell Int. 2021 May 3;21:247. doi: 10.1186/s12935-021-01918-8 (PMC8091726; doi:10.1186/s12935-021-01918-8)
Supplement: Supplementary file 2 — Additional file 2. Table S1 Details of the features of each tested platform, obtained from public data. Some information has been independently calculated/determined by the authors. Table S2 Image acquisition parameters for each platform for the plate, phantom and mice. Information in quotations is naming provided by software. Table S3 Statistics from Figure 1 EFG. Full statistical output from ANOVA, with Tukey comparisons. Table S4 Counts of tumours arising from tail vein injections, including percentages of the total number of tumours detected by each platform. The total tumour counts are listed in a separate table. [file 12935_2021_1918_MOESM2_ESM.docx]

**Supplementary Table 1**

|  | **Xenogen VivoVision IVIS 200** | **Bruker In-Vivo Xtreme (FI)** | **Li-Cor Pearl Trilogy** |
| --- | --- | --- | --- |
| Fluorescence | Yes | Yes | Yes |
| Luminescence | Yes | Yes | Yes |
| Light + Camera position | Above | Below (off centre) | Above |
| Illumination | Epi, Trans | Epi | Epi |
| Maximum number of mice per capture | 5 | 5 | 1 |
| Heating | Yes (shelf) | Yes (warmed air) | Yes (bed) |
|  |  |  |  |
| Lightsource (nm, fluorescence) | Xenon 350-1100 | Xenon 410-760 | Near-IR lasers |
| Imaging time (fluorescence) | Any | Any | 0.5 - 60s |
| Wavelengths fluorescence (nm) | 10 Ex filters 415-760 (+2) | 28 Ex filters (410-760) | Ex 685, Em 720 |
|  | 18 Em filters 490-850 (+6) | 6 Em filters (535-830) | Ex 785, Em 820 |
| FOV (cm) | 23 x 23 - 3.9 x 3.9 | 19 x 19 - 7.2 x 7.2 | 11.2 x 8.4 |
| Magnification range (x) | 1x - 8.7x | 1 - 2.6x* | 1 |
| Binning (n*n) | 4, 8, 16 | 1, 2, 4, 8, 16, 32 | 1, 2, 3 |
| Resolution without binning µm/pixel | 115 (full FOV) - <20 (zoomed)* | 93 (full FOV) - 35 (zoomed)* | 85 (full FOV) |
| Image acquisition | 16bit | 16bit | 22bit |
| Aperture | f/1 - f/8 | f/1.1 - f/16 | n.p.a. |
| Focal length | 50mm | 58mm | n.p.a. |
| Output | TIF 2048x2048 max | TIF 2048x2048 max | TIF 1300x964 max* |
| Software | Living Image® | Molecular Imaging | Image Studio^tm^ |

Notes:

* Determined/Calculated by authors

n.p.a.: not publicly available

**Supplementary Table 2**

| **plate** | **Xenogen VivoVision IVIS 200** | **Bruker In-Vivo Xtreme (FI)** | **Li-Cor Pearl Trilogy** |
| --- | --- | --- | --- |
| f-stop | f/1 | f/1.1 | n.p.a |
| time | 10s (cells) / 7s (antibody) | 30s (cells) , 10s (antibody) | n.p.a |
| Binning | “low” (4x4) | none | none |
| Excitation | "Cy5.5" (615-665 nm) | "690 nm" | 685 nm |
| Emission | "Cy5.5" (695-770 nm) | "750 nm" | 720 nm |
| Lamp level | "high" | n.p.a. | n.p.a. |
| Field of view | 13x13cm | 12 x 12 cm | 11.2 x 8.4 cm |

| **phantom** | **Xenogen VivoVision IVIS 200** | **Bruker In-Vivo Xtreme (FI)** | **Li-Cor Pearl Trilogy** |
| --- | --- | --- | --- |
| f-stop | f/1 | f/1.1 | n.p.a |
| time | 5s | 10s | n.p.a |
| Binning | “low” (4x4) | none | none |
| Excitation | "Cy5.5" (615-665 nm) | "690 nm" | 685 nm |
| Emission | "Cy5.5" (695-770 nm) | "750 nm" | 720 nm |
| Lamp level | "high" | n.p.a. | n.p.a. |
| Field of view | 13 x 13 cm | 12 x 12 cm | 11.2 x 8.4 cm |

| **mice** | **Xenogen VivoVision IVIS 200** | **Bruker In-Vivo Xtreme (FI)** | **Li-Cor Pearl Trilogy** |
| --- | --- | --- | --- |
| f-stop | f/2 | f/1.1 | n.p.a |
| time | 10s | 10s | n.p.a |
| Binning | “medium” (8x8) | none | 2x2 |
| Excitation | "Cy5.5" (615-665 nm) | "690 nm" | 685 nm |
| Emission | "Cy5.5" (695-770 nm) | "750 nm" | 720 nm |
| Lamp level | "high" | n.p.a. | n.p.a. |
| Field of view | 19.5 x 19.5 cm | 15 x 15 cm | 11.2 x 8.4 cm |

Notes:

n.p.a. not publicly available

**Supplementary Table 3**

| **XVI (Fig 1E)** |  |  |  |  |  |
| --- | --- | --- | --- | --- | --- |
| Tukey's multiple comparisons test | Mean Diff. | 95.00% CI of diff. | Below threshold? | Summary | Adjusted P Value |
| Day 17 vs. Day 21 | -7454950000 | -17350874014 to 2440974014 | No | ns | 0.1574 |
| Day 17 vs. Day 25 | -14804750000 | -24700674014 to -4908825986 | Yes | ** | 0.0039 |
| Day 21 vs. Day 25 | -7349800000 | -17245724014 to 2546124014 | No | ns | 0.1648 |
|  |  |  |  |  |  |
| **BIX (Fig 1F)** |  |  |  |  |  |
| Tukey's multiple comparisons test | Mean Diff. | 95.00% CI of diff. | Below threshold? | Summary | Adjusted P Value |
| Day 14 vs. Day 17 | -17118590 | -144304757 to 110067578 | No | ns | 0.9813 |
| Day 14 vs. Day 21 | -129382100 | -256568267 to -2195933 | Yes | * | 0.0453 |
| Day 14 vs. Day 24 | -226109597 | -353295764 to -98923430 | Yes | *** | 0.0004 |
| Day 17 vs. Day 21 | -112263510 | -239449677 to 14922657 | No | ns | 0.0957 |
| Day 17 vs. Day 24 | -208991008 | -336177175 to -81804841 | Yes | *** | 0.0009 |
| Day 21 vs. Day 24 | -96727497 | -223913664 to 30458670 | No | ns | 0.1782 |
|  |  |  |  |  |  |
| **LPT (Fig 1G)** |  |  |  |  |  |
| Tukey's multiple comparisons test | Mean Diff. | 95.00% CI of diff. | Below threshold? | Summary | Adjusted P Value |
| Day 14 vs. Day 17 | -2363 | -13146 to 8421 | No | ns | 0.9257 |
| Day 14 vs. Day 21 | -12570 | -23879 to -1260 | Yes | * | 0.0262 |
| Day 14 vs. Day 24 | -21603 | -32386 to -10819 | Yes | *** | 0.0001 |
| Day 17 vs. Day 21 | -10207 | -21517 to 1102 | No | ns | 0.0857 |
| Day 17 vs. Day 24 | -19240 | -30023 to -8457 | Yes | *** | 0.0004 |
| Day 21 vs. Day 24 | -9033 | -20342 to 2277 | No | ns | 0.1468 |
|  |  |  |  |  |  |
| **XVI (Fig 2G)** |  |  |  |  |  |
| Tukey's multiple comparisons test | Mean Diff. | 95.00% CI of diff. | Below threshold? | Summary | Adjusted P Value |
| Day 17 vs. Day 21 | -15.58 | -47.22 to 16.06 | No | ns | 0.4277 |
| Day 17 vs. Day 24 | -41.08 | -72.72 to -9.444 | Yes | * | 0.011 |
| Day 21 vs. Day 24 | -25.5 | -57.14 to 6.139 | No | ns | 0.1248 |
|  |  |  |  |  |  |
| **BIX (Fig 2H)** |  |  |  |  |  |
| Tukey's multiple comparisons test | Mean Diff. | 95.00% CI of diff. | Below threshold? | Summary | Adjusted P Value |
| Day 14 vs. Day 17 | -746.3 | -2632 to 1139 | No | ns | 0.689 |
| Day 14 vs. Day 21 | -2426 | -4311 to -539.8 | Yes | ** | 0.0089 |
| Day 14 vs. Day 24 | -3235 | -5121 to -1349 | Yes | *** | 0.0006 |
| Day 17 vs. Day 21 | -1679 | -3565 to 206.6 | No | ns | 0.0918 |
| Day 17 vs. Day 24 | -2489 | -4374 to -602.9 | Yes | ** | 0.0072 |
| Day 21 vs. Day 24 | -809.5 | -2695 to 1076 | No | ns | 0.633 |
|  |  |  |  |  |  |
| **LPT (Fig 2I)** |  |  |  |  |  |
| Tukey's multiple comparisons test | Mean Diff. | 95.00% CI of diff. | Below threshold? | Summary | Adjusted P Value |
| Day 14 vs. Day 17 | -336.5 | -1001 to 328.0 | No | ns | 0.5005 |
| Day 14 vs. Day 21 | -1655 | -2352 to -958.0 | Yes | **** | <0.0001 |
| Day 14 vs. Day 24 | -2424 | -3088 to -1760 | Yes | **** | <0.0001 |
| Day 17 vs. Day 21 | -1318 | -2015 to -621.5 | Yes | *** | 0.0002 |
| Day 17 vs. Day 24 | -2088 | -2752 to -1423 | Yes | **** | <0.0001 |
| Day 21 vs. Day 24 | -769.1 | -1466 to -72.23 | Yes | * | 0.0274 |

**Supplementary Table 4**

| **Platform** | Day 34 | Day 38 | Day 41 |
| --- | --- | --- | --- |
|  | **Number of tumours (%)** | **Number of tumours (%)** | **Number of tumours (%)** |
| XVI | 4 (28.6%) | 5 (35.7%) | 6 (42.9%) |
|  |  |  |  |
| BIX | 4 (28.6%) | 6 (42.9%) | 7 (50%) |
|  |  |  |  |
| LPT | 6 (42.9%) | 6 (42.9%) | 7 (50%) |
|  | | | |
| **Site** | | **Number of tumours identified *ex vivo*** | |
| Liver | | 6 | |
| Lungs | | 6 | |
| Limbs | | 2 | |
| Total | | 14 | |
